# Supplementary material for: MicroRNA and Protein Cargos of Human Limbal Epithelial Cell-Derived Exosomes and Their Regulatory Roles in Limbal Stromal Cells of Diabetic and Non-Diabetic Corneas
Source: Cells. 2023 Oct 25;12(21):2524. doi: 10.3390/cells12212524 (PMC10649916; doi:10.3390/cells12212524)
Supplement: Supplementary file 1 [file cells-12-02524-s001.zip › Supplementary Figure S1/Supplementary Figures.pdf]

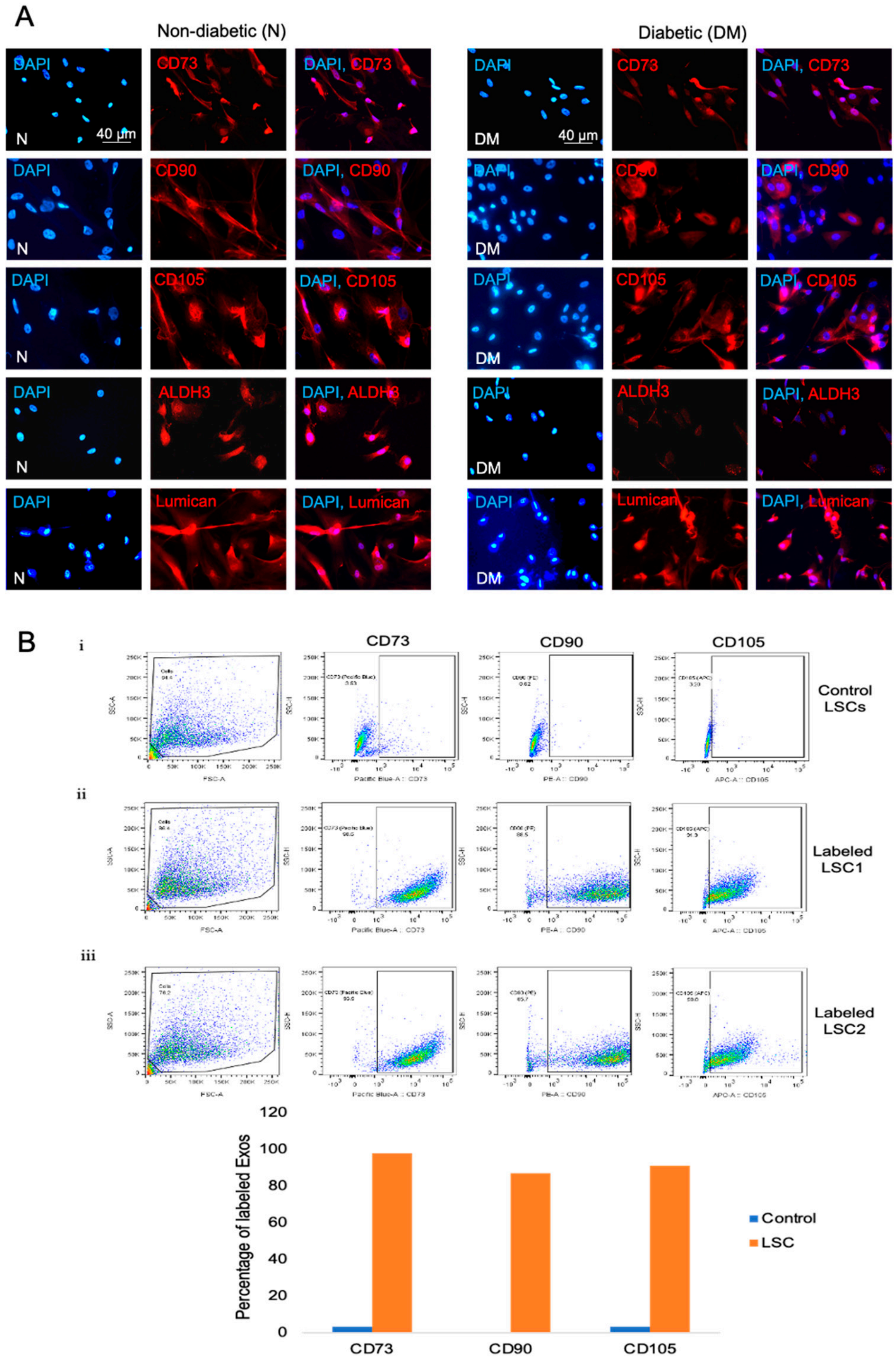

Supplementary Figure S1. Characterization of non-diabetic (N) and diabetic (DM) human primary cultured LSCs/LSSCs. **A**. A heterogenous population of limbal stromal cells showed expression of MSC markers such as CD73, CD90 and CD105, and keratocytes markers such as ALDH3A1 and Lumican in both N and DM. DAPI was used as a nuclear stain. **B.** Flow cytometry further confirmed the expression of CD73, CD90 and CD105 by non-diabetic LSSC. The bar graphs represent percent labeled cells *vs.* not labeled control cell population.
