# Supplementary material for: MicroRNA and Protein Cargos of Human Limbal Epithelial Cell-Derived Exosomes and Their Regulatory Roles in Limbal Stromal Cells of Diabetic and Non-Diabetic Corneas
Source: Cells. 2023 Oct 25;12(21):2524. doi: 10.3390/cells12212524 (PMC10649916; doi:10.3390/cells12212524)
Supplement: Supplementary file 1 [file cells-12-02524-s001.zip › Supplementary Table S1, S2.pdf]

Supplementary Table S1. Donor characteristics

**Case number (Years)**	**Age**	**Race**	**Gender**	**Cause of death**	**DM type /duration**	**History of eye diseases**
N19-53	69	Caucasian	M	Respiratory failure	N/A	None
N19-38	63	Caucasian	M	Cardiac arrest	N/A	None
N20-05	73	Caucasian	M	Shortness of breath	N/A	None
N20-14	80	Caucasian	F	Cardiopulmonary arrest	N/A	None
N20-12	77	Black	M	Cardiopulmonary arrest	N/A	None
N20-18	76	Caucasian	F	Cardiopulmonary arrest	N/A	None
N19-35	35	Caucasian	M	KCL overdose	N/A	None
N20-03	64	Caucasian	F	Cardiac arrest	N/A	None
N19-33	72	Caucasian	M	Cardiac arrest	N/A	None
N19-60	75	Caucasian	M	Colorectal cancer	N/A	None
N19-37	76	Black	M	Intraparenchymal hemorrhage	N/A	None
N20-21	73	Black	M	Natural passing	N/A	None
N20-14	80	Caucasian	F	Cardiopulmonary arrest	N/A	None
N20-02	76	Caucasian	M	Cardiopulmonary arrest	N/A	None
N20-15	78	Caucasian	M	Cardiovascular	N/A	Cataract
N23-03	80	Caucasian	M	Cardiac Arrest	N/A	None
N23-04	34	Caucasian	M	Choking	N/A	None
N23-13	62	Caucasian	M	Myocardial infarction	N/A	None
N23-14	60	Asian	F	Intracranial Hemorrhage	N/A	None
N23-15	77	Caucasian	F	Intracranial Hemorrhage	N/A	None
N23-21	74	Caucasian	M	Pneumonia	N/A	None
DM19-03	80	Caucasian	M	Heart failure	NIDDM /11	None
DM20-20	74	Caucasian	M	Cardiac arrest	NIDDM/30	Cataract
DM19-43	75	Caucasian	F	Cardiac arrest	NIDDM/30	None
DM20-01	77	Caucasian	F	Congestive heart failure	NIDDM/12	None
DM19-15	66	Caucasian	F	Decompensated NASH	NIDDM/5	None
DM19-07	75	Caucasian	F	Metastatic lung cancer	NIDDM/20	None
DM18-22	73	Caucasian	F	Cardiopulmonary arrest	NIDDM/40	None
DM18-29	58	Caucasian	M	Gunshot wound to chest	NIDDM/ 8	None
DM19-03	80	Caucasian	M	Heart failure	NIDDM/11	None
DM20-25	26	Caucasian	M	Anoxia	T1DM/15	None
DM23-06	74	Black	F	Congestive Heart failure	T1DM/20	Cataract
DM23-07	76	Caucasian	M	Myocardial infarction	T1DM/30	Corneal Scar
DM23-19	64	Caucasian	F	Anoxia Brain Injury	NIDDM/20	None

N, normal (non-diabetic); DM, diabetic mellitus; IDDM, T1DM; NIDDM, T2DM; M, male; F, female.

Supplementary Table S2. Primary antibody list

Antigen	Antibody	Source	Assay	Dilution
ALDH3A1	Mouse mAb LS-B14523 (clone 1B6)	LifeSpan BioSciences	WB, IF	1:1000, 1:100
CD73	Rabbit pAb PA5-11871	Invitrogen	WB, IF	1:500, 1:50
CD90	Rabbit mAb MA5-32559	Invitrogen	WB, IF	1:1000, 1:100
CD105	Rabbit pAb AF 1097	R&D Systems	WB, IF	1:500, 1:50
Keratocan	Rabbit pAb LS-C322431	LifeSpan BioSciences	WB	1:500
β-Actin	Mouse mAb A5316	Sigma-Aldrich	WB	1:1000
β-Actin	Rabbit mAb 8457	Cell Signaling Technology	WB	1:1000
CD63	Mouse mAb sc-5275	Santa Cruz Biotechnology	WB	1:500
CD63	Mouse mAb 353003 (clone H5C6)/PE	BioLegend	FC	1:100
CD81	Mouse mAb 349509 (clone 5A6)/APC	BioLegend	FC	1:100
HASP90	Mouse mAb Sc-24	Santa Cruz Biotechnology	WB	1:500
ERK1/2p44/42MAPK	Rabbit pAb cs-9102	Cell Signaling Technology	WB	1:500
ERK1(pT202/pY204 +ERK2(pT185/pY187)	Rabbit pAb ab4819	Abcam	WB	1:500
pAkt (Ser473)	Rabbit mAb 9271S	Cell Signaling Technology	WB	1:500
TSG101	Rabbit mAb ab125011	Abcam	WB	1:500

pAb, polyclonal antibody; mAb, monoclonal antibody; ALDH3,aldehyde dehydrogenase 3; WB, western blot; FC, flow cytometry; PE, Phycoerythrin (Fluorochrome), APC, Phycobilin pigments (Fluorochrome); IF, immunofluorescence.
