# Supplementary figures and images for: MicroRNA and Protein Cargos of Human Limbal Epithelial Cell-Derived Exosomes and Their Regulatory Roles in Limbal Stromal Cells of Diabetic and Non-Diabetic Corneas
Source: Cells. 2023 Oct 25;12(21):2524. doi: 10.3390/cells12212524 (PMC10649916; doi:10.3390/cells12212524)

Non-diabetic (N)

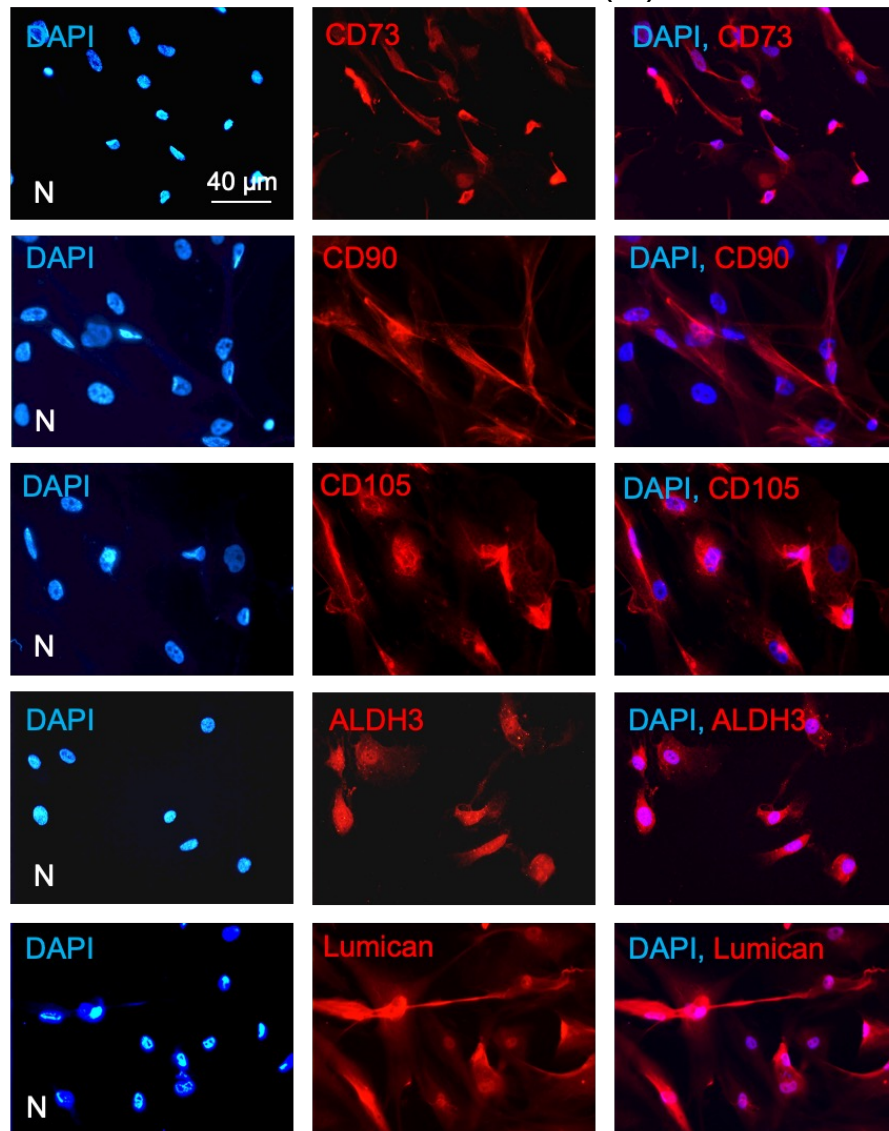

Diabetic (DM)

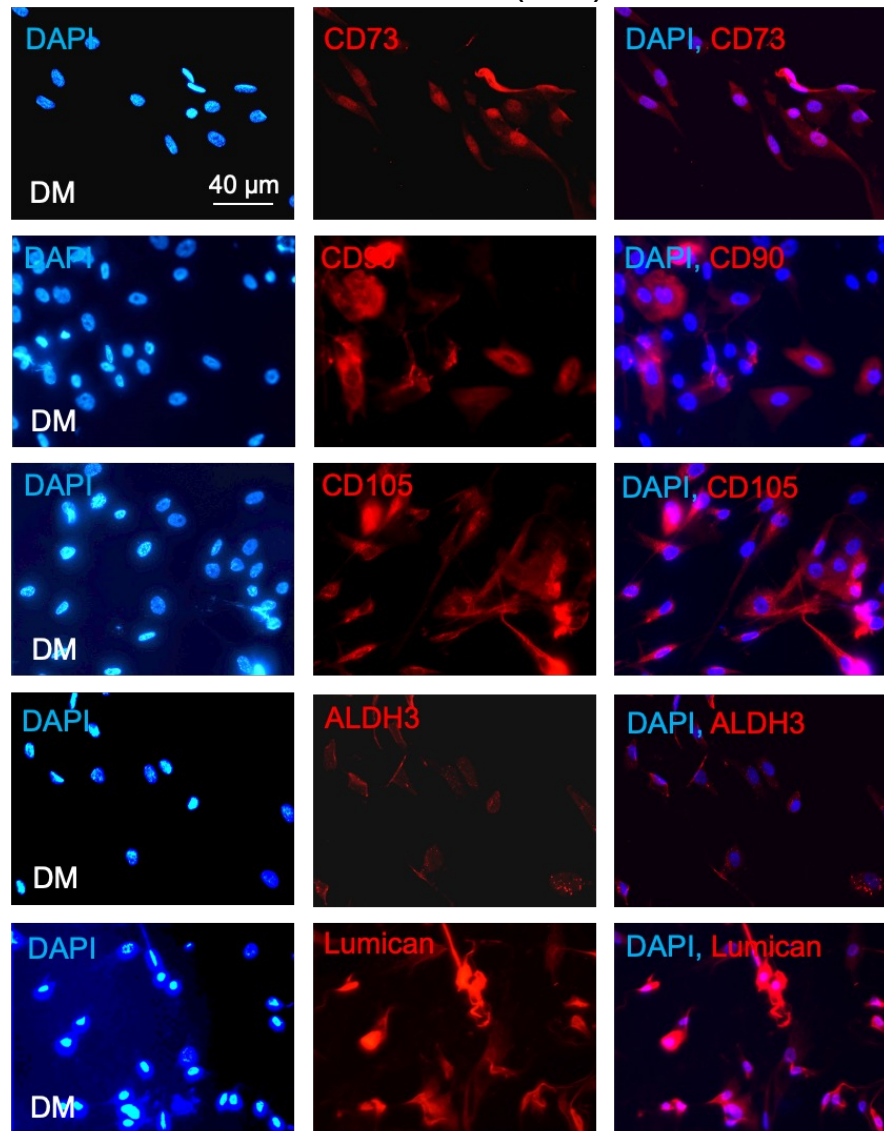

Supplement: Supplementary file 1 [file cells-12-02524-s001.zip › Supplementary Figure S1/Supplementary Figure S1A.pdf]

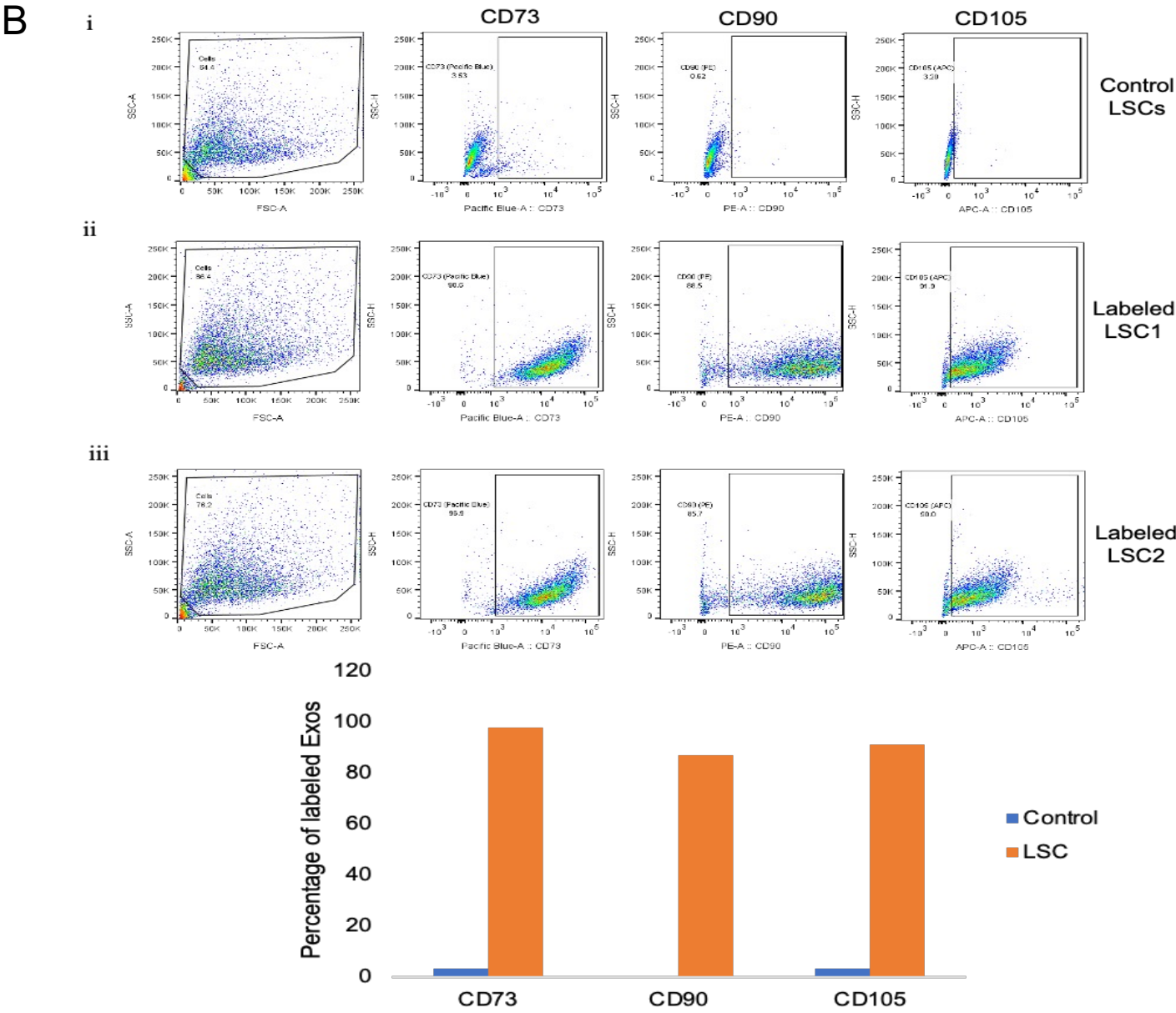

Supplement: Supplementary file 1 [file cells-12-02524-s001.zip › Supplementary Figure S1/Supplementary Figure S1B.pdf]
